# Urinary tract infection

**DOI:** 10.1186/1824-7288-40-S1-A17

**Published:** 2014-08-11

**Authors:** Claudio La Scola

**Affiliations:** 1 Paediatric Nephrology and Dialysis Unit, Department of Pediatrics, Sant’Orsola-Malpighi University Hospital, Bologna, 40138, Italy

## 

Urinary Tract Infections (UTIs) are common in every day pediatric practice. During the first 6 years of life, circa 6 to 7% of girls and 2.5% of boys will develop a UTI. UTI fall into two categories: non febrile lower UTI or cystitis and febrile upper UTI or pyelonephritis. The latter is considered by many to be the most common serious bacterial illness in children [1]. If UTI is suspected, urinalysis and urine culture should be performed. Collection methods used in clinical practice can be either invasive or non invasive, yet all carry a risk of contamination by bacteria not present in the bladder. Clean voided methods are preferable as they are quite easy to perform and reliable, while invasive methods should be limited to children in poor general health [2]. In the presence of clinical signs and positive urine analysis, while awaiting the results of antimicrobial sensitivity testing, antibiotic treatment should be started as soon as possible, considering local resistance patterns. The route of administration, either parenterally or orally, depends on the condition of the child and the severity of the infection and does not affect the duration of fever, recurrence of UTI or the incidence of post infectious scars. The need for imaging after a first febrile UTI has long been debated. New insights have led us to consider less aggressive imaging strategies, given the high rate of spontaneous resolution of vesico ureteral reflux with age and the good renal outcome for patients with acquired scarring. Therefore, voiding cystography and renal dimercaptosuccinic acid scintigraphy are not routinely recommended [2-4]. It is known that there are several risk factors for the recurrence of UTI. Some are not modifiable (age, gender, familiarity), while others (reflux, phimosis, bladder function, voiding habits, constipation and fluid intake) are modifiable thanks to behavioral changes and/or medical treatments. As regards preventive interventions, the most controversial is the use of antibiotic prophylaxis and currently none of the recently published guidelines recommend a routine use [2,4].
